# Synthesis, Characterization and Physicochemical Properties of Biogenic Silver Nanoparticle-Encapsulated Chitosan Bionanocomposites

**DOI:** 10.3390/polym14030463

**Published:** 2022-01-24

**Authors:** Sreelekha Ediyilyam, Mahesh M. Lalitha, Bini George, Sarojini Sharath Shankar, Stanisław Wacławek, Miroslav Černík, Vinod Vellora Thekkae Padil

**Affiliations:** 1 Department of Chemistry, School of Physical Sciences, Central University of Kerala, Kasaragod 671316, India; sreelekha.e469@gmail.com (S.E.); maheshml127@gmail.com (M.M.L.); 2 Department of Biochemistry and Molecular Biology, School of Biological Sciences, Central University of Kerala, Kasaragod 671316, India; 3 Department of Medicine, Thomas Jefferson University, Jefferson Alumni Hall, 1020 Locust Street, Philadelphia, PA 19107, USA; 4 Institute for Nanomaterials, Advanced Technologies and Innovation (CXI), Technical University of Liberec (TUL), Studentská 1402/2, 461 17 Liberec, Czech Republic; stanislaw.waclawek@tul.cz

**Keywords:** bionanocomposites, chitosan scaffold, silver nanoparticles, antioxidant, antibacterial, tween 80, glycerol, tissue engineering, bone regeneration, wound dressing

## Abstract

Green bionanocomposites have garnered considerable attention and applications in the pharmaceutical and packaging industries because of their intrinsic features, such as biocompatibility and biodegradability. The work presents a novel approach towards the combined effect of glycerol, tween 80 and silver nanoparticles (AgNPs) on the physicochemical properties of lyophilized chitosan (CH) scaffolds produced via a green synthesis method.The produced bionanocomposites were characterized with the help of Fourier transform infrared spectroscopy (FTIR) and Scanning electron microscopy (SEM). The swelling behavior, water vapor transmission rate, moisture retention capability, degradation in Hanks solution, biodegradability in soil, mechanical strength and electrochemical performance of the composites were evaluated. The addition of additives to the CH matrix alters the physicochemical and biological functioning of the matrix. Plasticized scaffolds showed an increase in swelling degree, water vapor transmission rate and degradability in Hank’s balanced solution compared to the blank chitosan scaffolds. The addition of tween 80 made the scaffolds more porous, and changes in physicochemical properties were observed. Green-synthesized AgNPs showed intensified antioxidant and antibacterial properties. Incorporating biogenic nanoparticles into the CH matrix enhances the polymer composites’ biochemical properties and increases the demand in the medical and biological sectors. These freeze-dried chitosan-AgNPs composite scaffolds had tremendous applications, especially in biomedical fields like wound dressing, tissue engineering, bone regeneration, etc.

## 1. Introduction

Over the last two decades, scientists have been more focused on polymer nanocomposites. Bionanocomposites are nanocomposites with at least one nanometer-scale dimension and are biologically existing polymers (biopolymer) bonded with an inorganic component [1]. They offer the notable merits of showing biocompatibility, biodegradability, and improved properties due to incorporated fillers or inorganic moieties. Depending on the nanofiller’s nature, the composite shows a modification in its mechanical, thermal, barrier and biological properties [2].

Chitosan (CH) is a deacetylated derivative of chitin, the world’s second most abundant polysaccharide after cellulose. It is a natural, biodegradable, biocompatible, non-toxic and antibacterial biopolymer that comes in several forms such as composites [3], hydrogel [4], film [5] and scaffolds [6]. CH scaffolds and derivatives have attracted significant interest in the biomedical field [7]. The employment of the chitosan scaffold alone is restricted by its poor mechanical and quick biodegradation characteristics. Still, when combined with a suitable modifier, it yields promising results by enhancing scaffold stability [8]. Thein-Han et al. [9] developed biodegradable chitosan-nanohydroxyapatite (nHA) scaffolds with superior physicochemical, biological, and mechanical properties as compared to chitosan scaffolds alone. CH scaffolds could also be used for potential therapeutic agent abstraction, controlled drug release [10], wound management [11], bone regeneration [12] and tissue engineering [13].

Polymer–nanoparticle composites have attracted the interest of researchers worldwide and have emerged as a critical topic of current study and development. It is due to the synergistic and hybrid qualities of fillers and matrices. Polymers are good filler hosting matrices for metal and metal oxide nanoparticles [14]. It has been found that integrating nanoparticles into a polymer inhibits aggregation and makes them more accessible. Recently, plant-mediated biologically prepared nanoparticles have obtained exceptional attention. It offers significant advantages in several areas, including simplicity of preparation, rate of formation, energy and safety management and capital expenditure. Silver nanoparticles (AgNPs) have been utilized to treat a variety of bacteria, including both Gram-positive and Gram-negative strains [15] and have an antioxidant and less toxic effect. Hence, they have been used to cure a variety of ailments. Owing to the antimicrobial properties of CH and AgNPs, CH-AgNPs composites have been used to produce medicinal products for the treatment of multidrug-resistant bacterial infections. For bone tissue engineering applications, freeze-dried nano TiO_2_/chitosan scaffolds were employed [16]. Sudheesh Kumar, P. T., et al. described the preparation of flexible and microporous chitosan hydrogel/nanoZnO composite bandages for wound dressing [17]. Plasticizers, such as glycerol, are commonly utilized to alter the mechanical characteristics of a film [18]. Plasticizers reduce intermolecular interactions between neighboring polymeric chains, boosting film flexibility, but they can also cause significant changes in the barrier properties of the film and help to ensure better dispersion of nanoparticles into the matrix [19]. Tween is a non-ionic emulsifier widely used in cosmetics, medicines and foodstuffs because of its efficacy at low concentrations and low toxicity [20]. There was a decrease in water vapor permeability values of scaffolds with the addition of surfactants due to the inclusion of components with specific hydrophobic properties [21].

The medicinal plant *Mussaenda frondosa* (*M. frondosa*) belongs to the family of Rubiaceae. It has traditionally been used to cure ailments like jaundice, ulcers, wounds, cough, bronchitis, white leprosy, eye problems, skin infections and tuberculosis [22]. Phytochemicals such as phenols, flavonoids, alkaloids, steroids, glycosides and tannins are abundant in all plants [23]. Chemical constituents identified in *M. frondosa* aqueous extracts include carbohydrates, tannins, alkaloids, flavonoids and polyphenols [24]. In- addition, the plant is frequently used in biomedical applications because of the presence of valuable and therapeutic bioactive components.

Our prior published study in the polymer journal has detailed the synthesis, characterization, antioxidant and antibacterial characteristics of AgNPs. The utilization of chitosan-gelatin-AgNPs as packaging materials was investigated. The synthesis, characterization, antioxidant and antimicrobial propertiesof AgNPs were also included in this study (Available online: https://doi.org/10.3390/polym13111680 (accessed on 21 May 2021).

The present work mainly focused on the preparation and different studies of biogenic AgNPs incorporated CH scaffolds. AgNPs were synthesized using *Mussaenda frondosa* plant extract; antioxidant and antibacterial activity was evaluated using DPPH and a broth dilution method. The lyophilization technique was employed for the preparation of chitosan–bionanocomposites. Mechanical, electrochemical, biodegradability and physiochemical properties of synthesized scaffolds were studied. Incorporating different moieties enhances the biological functioning and physicochemical properties; hence these biodegradable-biocompatible scaffolds can be used for several biomedical applications.

## 2. Materials and Methods

### 2.1. Materials

Silver nitrate (AgNO_3_), glycerol, tween 80 and glacial acetic acid were supplied from Merck chemicals, Mumbai, India. Hi-Media Laboratories Pvt. Ltd. (Mumbai, India) provided 90% deacetylated CH. Millipore water was used to make all aqueous solutions. *M. frondosa* leaves were collected from the Mulleriya forest in Kasaragod district, Kerala, India.

### 2.2. Methods

#### 2.2.1. Plant Extract Preparation

Shade-dried and powdered fresh leaves of *M. frondosa* were used.We mixed 10 g of the powdered specimen with 300 mL of Millipore water in an orbital shaker for 6 h. After being double-filtered using a muslin cloth, the extract was centrifuged. The plant extract obtained was subsequently lyophilized and utilized for future work.

#### 2.2.2. Synthesis of Silver Nanoparticles (AgNPs)

We added 1 mL of 10 mg/mL M. frondosa leaf extract to 10 mL of 10 mM AgNO3 solution to make AgNPs. The obtained solution was stirred at 80 °C for 1 h in a magnetic stirrer. The generation of AgNPs was understood by the color shift from pale yellow to reddish-brown (Figure S1). This colloidal solution was lyophilized and used in later experiments.

#### 2.2.3. Preparation of CH Scaffolds

We dissolved 2 g of CH in 1% acetic acid solution at room temperature to make the CH solution. The undissolved particles were separated from the resulting solution mixture by filtration. The ensuing hydrogel was neutralized by adding a 1% NaOH solution. The additives glycerol, tween 80 and AgNPs, were added to the CH solution mixture based on CH weight and composition of different additives shown in Table 1. The CH mixture was maintained at room temperature for 1 h inside an orbital shaker. The homogenized sample was placed into a Teflon tray and chilled overnight at −80 °C. The frozen specimens were lyophilized for 24 h and produced chitosan scaffolds.

### 2.3. Characterization

The optical characteristics of AgNPs were measured by recording UV-visible absorption spectra over the spectral range of 200–800 nm on a Perkin Elmer UV-Vis Spectrometer Lambada-35 (Perkin-Elmer SCIEX, Waltham, MA, USA). The spectrometer Perkin Elmer Spectrum Two (Perkin-Elmer SCIEX, Waltham, MA, USA) was used for attenuated total reflection (ATR) mode at 400–4000 cm^−1^ for Fourier transform infrared analysis. The XRD pattern was acquired using a nickel-filtered Cukα radiation source (λ = 0.154056 nm) radiation and a liquid nitrogen-cooled germanium solid-state detector using an X-ray diffractometer (XRD, Rigaku Miniflex600, Tokyo, Japan) over the diffraction angle (2θ scale) operating range from 10° to 90°. High-resolution transmission electron microscopy (HR-TEM) was used to obtain the crystalline structure, morphology and particle size distributions of AgNPs (JEOL, JEM-2100, Tokyo, Japan) operated at an accelerating voltage of 200 kV with the resolution point 0.23 nm and lattice 0.14 nm.CH composites were characterized using FTIR (Perkin-Elmer SCIEX, Waltham, MA, USA) and SEM (JEOL, JSM-6490LA, Tokyo, Japan). The tensile strength and elongation at break of the scaffolds were obtained with a Universal Testing Machine (Shimadzu AG-Xplus) following the ASTM standard method D882. The average thickness of scaffolds was determined by measuring the thickness at six distinct points using a digital micrometer. An electrochemical analyzer was used to conduct cyclic voltammetric (CV) tests (CHI 1108; CH Instruments, Bee Cave, TX, USA). A three-electrode cell system consists of an Ag/AgCl reference electrode and platinum counter electrode. CH-AgNPs solution was prepared by adding 0.2 g of AgNPs to the 100 mL of 2% CH solution. The modified electrode was made by dropping 2 μL of CH-AgNPs or CH solution over the clean surface of bare glassy carbon (GC) and allowing it to dry. All electrochemical studies were then carried out using modified/bare electrodes as the working electrode and an Ag/AgCl (in saturated KCl) electrode as the reference electrode. Cyclic voltammograms of 0.1 M ferricyanide in 1 M KCl were used to assess the electrochemical performance of the bare/modified electrode.

### 2.4. Evaluation of the Antioxidant Activity of AgNPs Using the DPPH Radical Scavenging Method

Green synthesized AgNPs (5 µg/mL to 200 µg/mL) were combined with 1 mL of 0.01 mM DPPH solution. After properly mixing and resting at room temperature for about 15 min, the absorbance of various samples was measured using a spectrophotometer at 517 nm. The % of the DPPH scavenging effect was calculated using the formula given below.

$$\%\;\mathrm{of}\;\mathrm{inhibition}=\frac{\mathrm{Absorption}\;\mathrm{of}\;\mathrm{control}-\mathrm{Absorption}\;\mathrm{of}\;\mathrm{test}}{\mathrm{Absorption}\;\mathrm{of}\;\mathrm{control}}\times 100$$

### 2.5. Antimicrobial Activity of AgNPs

Two-fold serial dilution procedures were used to determine the minimal inhibitory concentration (MIC). Stock inoculums growth was adjusted to 1% McFarland Standard. In 96-well microtiter plates, the broth dilution assay was performed.100 μL of diluted conidial inoculums solutions were added to each well in the plate, and the final volume was adjusted to 200 μL. The samples were weighed (1 mg), dissolved in DMSO to get a stock solution of 1 mg/mL, and then added to the wells in decreasing concentrations of 62.5, 31.25, 15.63, 7.81, 3.90 µg/mL, respectively, before being incubated overnight at room temperature. A control well was kept with the organism alone and visual inspection and optical density (OD) measurements were takenat 630 nm using an ELISA plate reader. The OD was taken a right after the visual reading. The following formula was used to calculate the growth inhibition for the test wells at each extract dilution:

$$\mathrm{Percentage}\;\mathrm{of}\;\mathrm{inhibition}=\frac{\left(\mathrm{OD}\;\mathrm{of}\;\mathrm{control}-\mathrm{OD}\;\mathrm{of}\;\mathrm{test}\right)}{\left(\mathrm{OD}\;\mathrm{of}\;\mathrm{control}\right)\times 100}$$

### 2.6. Swelling Degree (%W)

The disparity in weights of the dried scaffolds (Wd) and the weights of the scaffolds after soaking in water (Ws) for 24 h at 37 °C and wiping out surface water using tissue paper were used to assess the scaffolds’ swelling degree. The equilibrium degree of swelling EDS (%) was determined using the equationbelow:

$$\mathrm{EDS}\;\left(\%\right)=\frac{\mathrm{Ws}-\mathrm{Wd}}{\mathrm{Wd}}\times 100$$

Ws—Initial weight of the scaffold sample (g).
Wd—Final weight of the scaffold sample (g).

### 2.7. Water Vapor Transmission Rate (WVTR)

We poured 10 mL deionized water into 1.5 cm mouth diameter bottles. Polymer scaffolds were used to cover the top opening of the bottle and tape. The weight of the bottle with water was taken and placed in an oven for 24 h at 40 °C. The bottle was kept out from the oven after 24 h and reweighed. The following equations were used to calculate the WVTR of the scaffolds {g/m^2^ h}.

$$\mathrm{WVTR}=\frac{W_{i}-W_{t}}{A\times t}$$

W_i_—Initial weight of scaffold sample (g).
W_t_—Final weight of scaffold sample (g).
A—Mouth area of the bottle (m^2^).
t—Time in h.

### 2.8. Test for Degradation in Hank’s Solution

The goal of this study was to see how far scaffolds degraded by immersing them in a synthetic body fluid (Hank’s solution) with an ionic strength similar to human blood plasma. Hank’s solution was made by combining NaCl (8 g), NaHCO_3_ (0.35 g), KCl (0.4 g), KH_2_PO_4_ (0.06 g), MgCl_2_·6H_2_O (0.10 g), CaCl_2_ (0.14 g), Na_2_HPO_4_ (0.06 g), MgSO_4_·7H_2_O (0.06 g) and glucose (1 g) in 1000 mL of water.

Dry scaffold samples were soaked in Hank’s solution for one week before being dried and weighed. After immersion, the scaffold weight change was used to track the degree of deterioration. The scaffolds’ net weight loss or % of decay (% D) was computed as follows:

$$\%\;\mathrm{of}\;D=\frac{D_{2}-D_{1}}{D_{1}}\times 100$$

D_2_—Weight of the scaffolds after one week (g).
D_1_—Dry weight of the scaffolds sample (g).

### 2.9. Test of Biodegradability

The test samples were buried for1week in a container containing soil at a depth of 10 cm to simulate soil burial deterioration. The soil’s moisture level was sustained by splashing water at regular intervals. Excess water was emptied from the pot through a hole in the bottom. The deterioration of the samples was measured at regular intervals by carefully extracting the soil sample and gently washing it. Over time, the weight difference or loss indicated the soil burial degradation rate.

The weight loss was determined using the below equation,

$$\mathrm{Weight}\;\mathrm{loss}=\frac{W_{f}-W_{d}}{W_{f}}\times 100$$

W_f_—Final weight of the scaffold (g).
W_d_—Dry weight of the scaffold (g).

### 2.10. Moisture Retention Capability (MRC)

Scaffolds of almost the same thickness were cut and weighed to assess moisture retention capacities. These scaffolds were kept for 6 h at 40 °C.; the moisture retention capacity was determined using the following formula:

$$\mathrm{MRC}=\frac{W_{t}}{W_{i}}\times 100$$

W_t_—Weight of scaffold specimen at time t (g).
W_i_—Initial weight of the scaffold specimen (g).

## 3. Results and Discussions

### 3.1. Characterization of AgNPs

#### 3.1.1. Ultraviolet (UV)–Visible Spectroscopy and Transmission Electron Microscopy (TEM) Analysis

UV-Visible data wereused to verify the generation and stability of the AgNPs. It was also utilized to determine the reaction progress and completion. The characteristic absorption peak of AgNPs ranged from 390–470 nm, and green synthesized AgNPs showed an absorption peak at 426 nm. This is primarily validated the formation of AgNPs [3]. TEM micrographs of biogenic AgNPs showed that most of the synthesized nanoparticles were spherical in morphology. Triangle and quasi-spherical nanoparticles were also identified. The nanoparticles generated utilizing a biological system had many shape and size variations. The particle size was estimated to be between 10–30 nm. Lattice fringes with an interplanar spacing of 0.24 nm were seen in the HRTEM (high-resolution TEM) image ascribed to (111) planes of AgNPs. The selected area electron diffraction pattern (SAED) showed concentric rings, suggesting the crystalline nature of AgNPs [3]. (Available online: https://doi.org/10.3390/polym13111680 (accessed on 21 May 2021).

#### 3.1.2. X-ray Diffraction Studies

**Figure 1:**
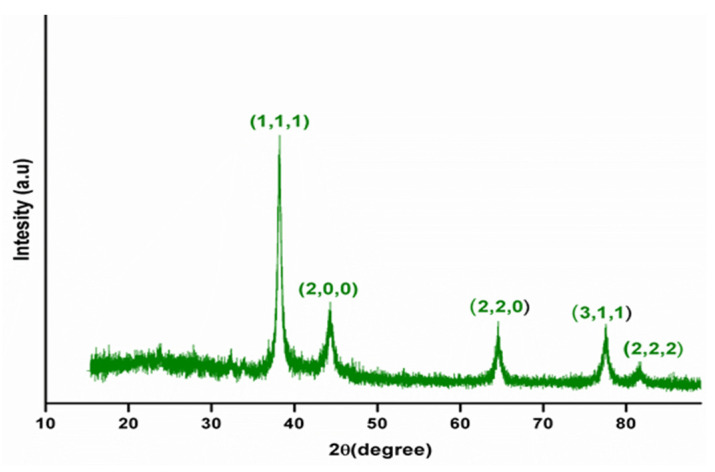
X-ray diffraction patterns of green synthesized silver nanoparticles (AgNPs) using *M. frondosa* leaf extract.

The crystalline nature, phase formation and purity of the synthesized nanoparticles were determined using XRD analysis. Figure 1 depicts the XRD spectra of biogenic AgNPs. XRD analysis was carried out by heating a lyophilized sample at 80 °C for 3 h. Diffraction peaks were observed in green synthesized nanoparticles at 38.22°, 44.74°, 64.73°, 77.7° and 81.78°, which were correlated to the (111), (200), (220), (311) and (222) reflection planes of metallic AgNPs’ face-centered cubic structure. The lattice parameters of AgNPs perfectly agreed with the standard JCPDS file No. 04-0783 [25].

### 3.2. Antioxidant Activity of AgNPs

1,1-Diphenyl-2-picrylhydrazl (DPPH) is a purple-colored strong oxidant with significant absorption maxima at 517 nm. In the presence of an antioxidant, the free radical in DPPH is coupled off. As a result, the absorption and color intensity gets reduced. The DPPH radicals were reduced when AgNPs gave them an electron or proton. When the nanoparticle concentration was raised from 5 µg/mL to 200 µg/mL, the percentage of scavenging activity increased steadily. Green-synthesized AgNPs exhibited 31% scavenging activity at 5 µg/mL concentration and 69% scavenging activity at 200 µg/mL concentration. Due to the occurrence of a biologically active agent on the surface of the nanoparticles made from *M. frondosa* leaf extract, it had higher antioxidant activity. Flavonoids and phenolic substances possess potent antioxidant activities, preventing and treating degenerative diseases [3]. (Available online: https://doi.org/10.3390/polym13111680 (accessed on 21 May 2021)).

### 3.3. Antimicrobial Activity

**Figure 2:**
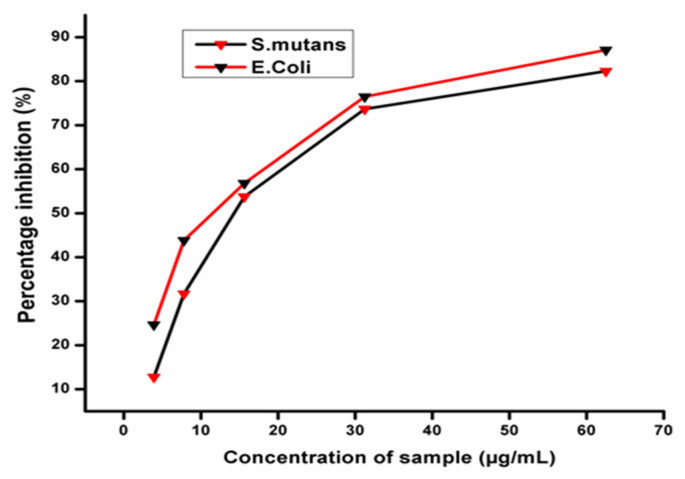
Antibacterial activity of AgNPs.

The bactericidal activity of AgNPs produced was tested against Gram-negative *Escherichia coli* (*E. coli*) and Gram-positive *Streptococcus mutans* (*S. mutans*) and dose-dependent relationships were discovered (Figure 2). For each bacterial species, the AgNPs’ MIC values were computed. These findings also showed that bacterial inhibition increased with the concentration of AgNPs. The MICs (IC50) of AgNPs produced against *E. coli* and *S.mutans* were 12.12 µg/mL and 20.47 µg/mL, respectively. A comparison of the obtained inhibitory activity indicated that AgNPs synthesized using *M. frondosa* extract were more active against the Gram-negative bacteria *E. coli* than Gram-positive *S. mutans*. Earlier research on the interaction of AgNPs with *E. coli* confirmed that AgNPs adhere to the bacterial cell wall during the initial step of the interaction. After establishing a stable adhesion, AgNPs penetrate into the bacteria and rupture the cell membrane, causing cell death. AgNPs acting as oxidizing agents on the surface of plasma membrane proteins and cellular homeostasis have also been proposed as mechanisms for AgNPs antibacterial activity [26]. Another notion is that AgNPs bind to the surface of the cell membrane, reducing permeability and respiration [27]. Plant-synthesized AgNPs have a benefit over chemically synthesized AgNPs regarding the ability of phytoconstituents to act as capping and stabilizing agents and have anantimicrobial effect of their own, which enhances AgNPs’ antibacterial activity.

### 3.4. Characterization of CH Scaffolds

**Figure 3:**
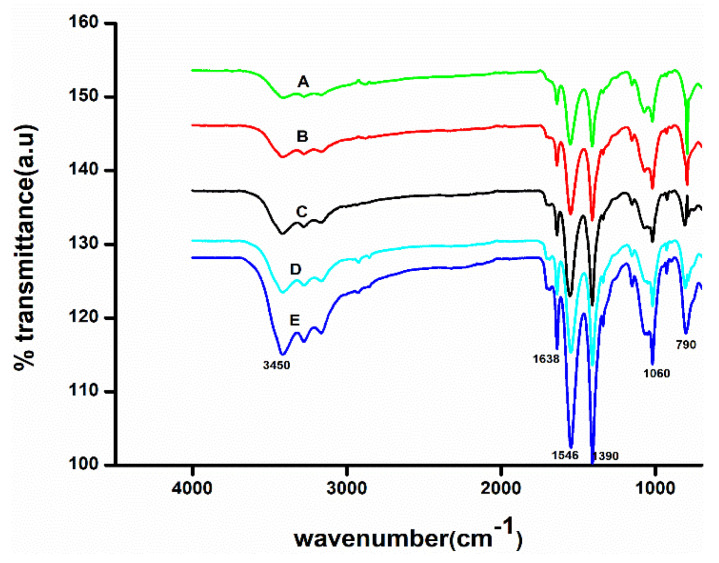
Fourier transform infrared (FTIR) spectra of (A) CH scaffolds (Cp) (B) CH plasticized scaffolds (Cp1) (C) CH plasticized- and surfactant-added scaffolds (Cp2) (D) Cp2+.002 AgNPs (Cp5) (E) Cp2+.0025 AgNPs (Cp6).

FTIR spectra of CH composites showed all the characteristic peaks of CH (Figure 3). Accordingly, the 1060 cm^−1^ corresponds to C-O-C stretching, the bands at 1638 cm^−1^ and 1546 cm^−1^ for amide I and amide II bands [21]. The FTIR band at 790 cm^−1^ indicates C-H deformation. The FTIR peak at 1390 cm^−1^ is N-H stretching or C-N bond stretching vibration [28]. It showed broadband at 3450 cm^−1^ corresponding to amine N-H symmetrical vibration or H bonded O-H group stretching vibration [29]. Upon the addition of glycerol, tween 80 and AgNPs did not have any considerable change in IR spectra. The additives did not affect the chemical structure of CH. There was no formation of a new bond when additives were added; however, the IR band’s intensity increased [21].

**Figure 4:**
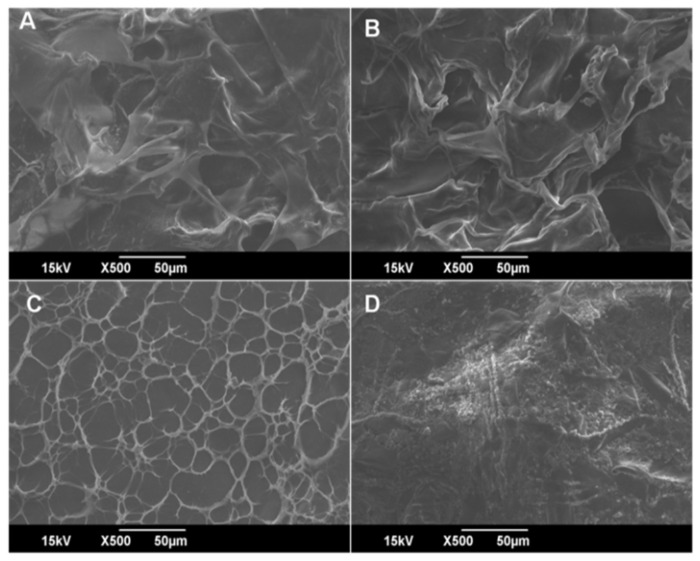
Scanning electron microscopy (SEM) images of (**A**) CH scaffolds (Cp) (**B**) CH plasticized scaffolds (Cp1) (**C**) CH plasticized- and surfactant-added scaffolds (Cp2); (**D**) Cp2+0.0025 AgNPs (Cp6).

Scanning electron microscopy (SEM) was used to investigate the surface morphology of CH scaffolds and is displayed in Figure 4A–D. Surface structures on the CH were homogeneous, dense and smooth. SEM micrographs show that the solvent primarily determines the shape of the scaffold. The solvent has a more significant influence on the morphological features of the scaffolds and the inclusion of additives has an impact, but only as a secondary factor. Due to the apparent plasticizer effect, the presence of glycerol on the scaffold produces a more homogeneous surface than without glycerol [30]. The presence of tween 80 generates some additional porosity on the scaffolds, as shown in Figure 4C [31]. The uniform dispersion of nanoparticles caused compact and dense scaffolds with decreased porosity after adding AgNPs. Nevertheless, some aggregation of nanoparticles was observed (Figure 4D), which could be attributed to the presence of the capping agent and the surface was also quite rough. It is worth mentioning that the particles were not uniformly mixed in a CH matrix [32].

### 3.5. Electrochemical Performance of the Fabricated CH-Ag NPS/GC (Chitosan-Silver Nanoparticles Coated on Glassy Carbon) in a Ferri/Ferro Probe

**Figure 5:**
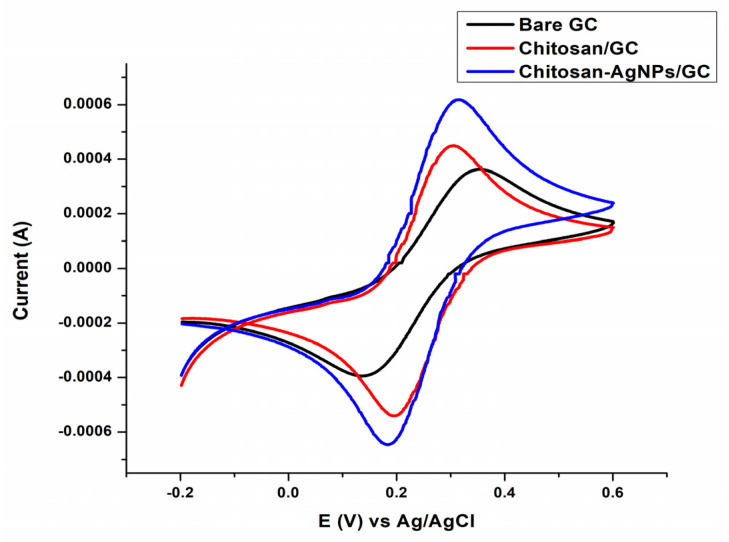
Cyclic voltammograms of the GC (Bare glassy carbon), CH/GC (chitosan modified glassy carbon) and CH-AgNPs/GC (chitosan-Silver nanoparticles modified glassy carbon) in 0.1 M ferricyanide solution in 10 mL of 1 M KCl with a scan rate of 1 V/s.

To investigate the electrochemical behavior of the bare/modified electrode, a cyclic voltammogram of 0.1 M ferricyanide in 1 M KCl supporting electrolyte was performed at a scan rate of 1 V s^−1^. Figure 5 illustrates a detailed cyclic-voltammogram of 0.1 M K4 [Fe (CN) 6] on bare glassy carbon (GC), chitosan modified glassy carbon (CH/GC) and chitosan-silver nanoparticles-modified glassy carbon (CH-AgNPs/GC). A reversible voltammogram was obtained on the GC, with cathodic peak current (Ipc) of 359 µA and anodic peak current (Ipa) of 400 µA, corresponding to potentials of 0.357 and 0.140 V. CH-modified GC shows the enhanced oxidation and reduction peak currents of the 444 and 541 µA, corresponding to a potential of 0.304 and 0.200 V. However, the modified electrode CH-Ag NPS/GC showed the most increased anodic and cathodic peak current values, i.e., 606 and 646 µA, corresponding to the potentials 0.312 and 0.182 V. These findings showed that the CH-Ag NPS/GC modified electrode has improved electrocatalytic capabilities towards the electrochemical probe. According to Shu, Yun et al., the background current of MnO_2_ nanosheet/GCE was greater than that of the bare glassy carbon electrode (GCE). Therefore, a modified MnO_2_ nanosheet/GCE was used for the electrochemical detection of H_2_O_2_ [33]. Sivan, SaranyaKothaplamoottil, et al. described the enhancement of reduction peak current of TiO_2_NPS/CPE (TiO_2_ nanoparticles modified the surface of carbon paste electrode). This modified electrode demonstrated increased electrocatalytic activity towards the electrochemical probe; it was utilized to detect heavy metal Pb^2+^ ions in plastic toys [34].

### 3.6. Evaluation of Mechanical Properties

Mechanical properties of CH scaffolds are represented in Table 2. The pure CH scaffold has a TS of 0.96 MPa and the % of EAB is 4.63. The addition of glycerol to the CH significantly lowered the scaffolds’ mechanical strength and modulus. Although the reverse tendency was found for % of EAB, it rose with the glycerol amount. When glycerol is added to the CH matrix, intermolecular attraction decreases and facilitates polymer mobility, ultimately leading to the rise of % of EAB [35]. Glycerol-added composites had reduced tensile strength due to the increased amorphous nature in the composites [36]. When the surfactant tween 80 was added, we noticed a synergistic effect between glycerol and tween 80 surfactants. The presence of surfactants caused an increase in tensile strength but a reduction in % of EAB. The increase in tensile strength could be due to the inclusion of hydrophobic features of surfactants [37]. The addition of surfactant lowered the plasticizing effect of glycerol; hence there was a reduction of elongation at break compared to the glycerol CH composites [38]. The incorporation of AgNPs resulted in a slight variation of tensile strength and % of EAB.

### 3.7. Swelling Ratio of CH Scaffolds and Water Vapour Transmission Rate (WVTR)

**Figure 6:**
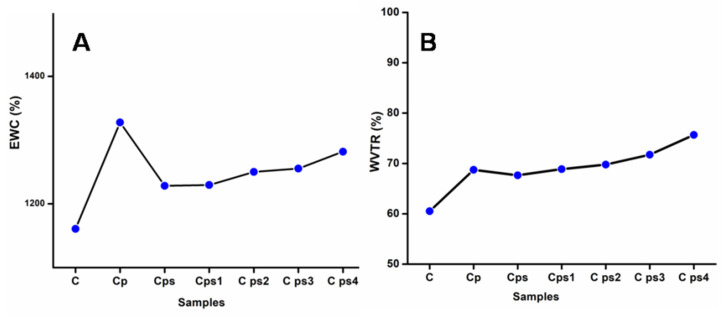
(**A**) Equilibrium degree of swelling of CH scaffolds (**B**) Water vapor transmission rate of CH scaffolds.

The swelling ratio of CH scaffolds is represented in Figure 6A. The swelling is characterized as a change from an unsolvated to a swollen (solvated) condition of the hydrogel nanocomposite pores. The solution diffusion into the hydrogel’s polymeric matrix results in swollen and external fluid retention. Incorporating plasticizers into the CH generates flexibility and usually increases the swelling degree of scaffolds [36]. The addition of glycerol improved the composites’ dimensional stability. The attractive forces between anionic polymeric chains and cationic surfactant molecules increase with the addition of surfactants, resulting in physical cross-linking in the polymer networks, which reduces free space in the network and slows water molecule migration into the hydrogel [39]. Therefore, the surfactant tween 80 decreases the swelling degree of scaffolds. The surface of green synthesized nanoparticles stabilized with hydrophilic phytochemicals like carbohydrates, tannins, alkaloids, flavonoids and polyphenols present in the *M. frondosa* leaf extract [11], which is mainly associated with the reduction of nanoparticles. Water-soluble biomolecules coated on the surface of AgNPs stabilize the nanoparticle and increase the swelling degree of scaffolds. The swelling ratio of gelatin/carboxymethyl chitosan/LAPONITE composite scaffold was in agreement with our findings [40].

The pace at which water vapor permeates through a substance is called the water vapor transmission rate (WVTR), as shown in Figure 6B. The mechanisms for predicting water transport through hydrophilic material are incredibly complex. When the films are cationic and fully hydrophilic, water mesh with the polymer matrix increases water vapor permeation. The pervading molecules condense on the surface and solubilize into the film and propagate through the film [41]. Finally, these molecules cross the film’s surface to the other side. When plasticizer glycerol is connected to the CH matrix, the water permeability of the composite increases. Glycerol decreases intermolecular attraction between adjacent polymer chains and leads to water migration from the polymer matrix [42]. The water vapor transmission rate was unaffected by the presence of surfactants in scaffolds without glycerol. The HLB value of tween 80 is 4.3 and it has hydrophobic characteristics. We integrated components with some hydrophobic elements to the matrix with the addition of surfactants, so water vapor permeability was expected to reduce [36]. When nanoparticles were added to the scaffolds, they became more flexible and acted as plasticizers; so WVTR increased. Shi Yufei et al. described the study of two porous sponge scaffolds prepared by collagen derived from porcine skin and fish scales as burn wound dressings. WVTR of these scaffolds was very high; these materials were excellent for wound-healing applications [43].

### 3.8. Moisture Retention Capability (MRC) and Degradation in Hank’s Solution

**Figure 7:**
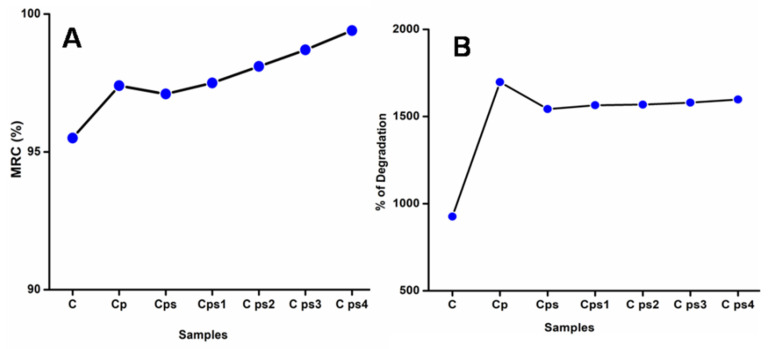
(**A**) Moisture retention capability of CH scaffolds (**B**) Degradation of CH scaffolds in Hank’s solution comprisinginorganic ions and glucose.

The amount of water vapor lost from the films is governed by moisture retention capacity. Results indicated the high moisture retention capability of scaffolds shown in Figure 7A. There were no significant variations of MRC values amongst the composites after 6 h, with a range of 95–97%, indicating that the composites had almost identical moisture retention performance independent of their compositions. Glycerol-plasticized CH scaffolds contain highermoisture content than other scaffolds and the increase in glycerol amount dramatically increases the moisture contents of the scaffolds [44]. The hydrophilic property of glycerol was linked to the better moisture retention capacities of glycerol-plasticized CH scaffolds. Glycerol’s OH groups can retain water within nanocomposite scaffolds. Glycerol’s hydroxyl groups significantly attract water molecules, allowing scaffolds to retain water and quickly create hydrogen bonds inside their matrix. As a result, thisworks as a water-holding agent and is critical to composites’ exceptional moisture retention capability [45]. The scaffolds’ moisture retention capability slightly decreased by adding nanoparticles due to the destruction of the composite material’s network structure [46].

Degradation in Hank’s solution is represented in Figure 7B. Hank’s balanced salt solution is made up of inorganic salts with the addition of glucose. It maintains physiological pH and osmotic pressure. The possibility of applying these porous scaffolds for tissue engineering and wound healing is unquestionably an added worth to be considered. The scaffolds’ hydrophilicity and swelling degree influence the degradation of scaffolds in Hank’s solution [47]. The addition of plasticizers and surfactants accelerates the rate of deterioration in Hank’s balanced salt solution; Vicentin et al. reported similar results for surfactant- and plasticizer-added PVA/CH films [31]. With the addition of AgNPs, the degradation of scaffolds in Hank’s balanced salt solution increased [24].

Mean values, standard deviation and standard error of swelling degree, water vaportransmission rate, moisture retention capabilityand degradation in Hank’s solution, % of EAB and TS of CH scaffolds are calculated and included in supplementary data (Tables S1–S6). 

### 3.9. Biodegradability of CH Scaffolds

**Figure 8:**
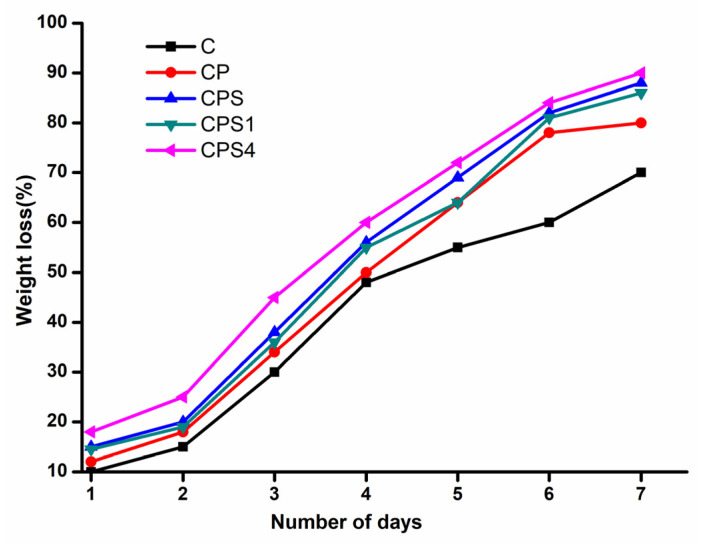
Soil biodegradability of CH scaffolds at different intervals of days.

After 7 days of exposure to the soil under existing environmental conditions, the CH scaffolds shrank in size and appeared hard and brittle. After soil exposure, scaffold degradation was followed by reducing overall weight loss at various time intervals to determine the degradation rates, as illustrated in Figure 8. The pure CH scaffolds lost most of their weight after 7 days, owing to the composites’ hydrophilic characteristic, allowing moisture from the soil to readily infiltrate the polymer structure, degrading the polymer chains and making them prone to microbial destruction [48]. It was observed that the degradation of AgNPsadded scaffolds was slow compared to other scaffolds [3]. It may occur due to the antibacterial potential of AgNPs [49].

## 4. Conclusions

AgNPs with particle sizes ranging from 10–30 nm were synthesized employing *M. frondosa* leaf extract and AgNO_3_ solution. The antioxidant and antibacterial properties of green synthesized AgNPs were remarkable. Plasticizer glycerol, surfactant tween 80 and AgNPs were added to the CH matrix and freeze-dried to produce CH scaffolds.IR curves were not affected by the presence of glycerol, tween 80 and AgNPs, as evidenced by the lack of new kinds of bonds in IR spectra. The homogeneous distribution of AgNPs within the CH matrix can be seen in SEM micrographs. This also demonstrates that the addition of tween 80 increases porosity. The synthesized composites have almost similar MRC values of 95–97% regardless of their compositions. The swelling degree increased when glycerol and AgNPs were added to the CH matrix. However, due to the physical cross-linking in polymer networks, the addition of tween 80 to the matrix resulted in the reduction of swelling. WVTR was enhanced by incorporating glycerol and AgNPs, but the addition of the surfactant tween 80 led to a modest reduction of it. All composites showed better degradation in Hank’s balanced salt solution. Mechanical strength of composites slightly changes upon the addition of plasticizers, surfactants and AgNPs. The entire scaffold’s size was diminished in soil, demonstrating the natural biodegradation in the soil environment. CH showed the highest percentage of biodegradation among the scaffolds by soil microorganisms. AgNP-added scaffolds showed negligible degradation, which may be due to the antimicrobial action of AgNPs. The results demonstrate that the prepared lyophilized CH composites can be used mainly for biomedical applications.

Further research is still required to improve the quality of life and understand the full potential of bionanocomposites in biomedical fields; the future also focuses on the cost-effective production of more sustainable materials. Even though there is tremendous growth in the bionanocomposite field, the transformation from the smaller-scale laboratory to the larger-scale industry requires constant development and innovation in the material science and manufacturing field.

## Figures and Tables

**Table 1:** Composition of CH composites.

Sample ID	Chitosan (CH) (% *w*/*v*)	Glycerol (% *w*/*v*)	Tween 80 (% *w*/*v*)	Silver Nanoparticles (AgNPs) (% *w*/*v*)
Cp	2	0	0	0
Cp1	2	0.05	0	0
Cp2	2	0.05	0.05	0
Cp3	2	0.05	0.05	0.001
Cp4	2	0.05	0.05	0.0015
Cp5	2	0.05	0.05	0.002
Cp6	2	0.05	0.05	0.0025

**Table 2:** Mechanical properties of CH scaffolds.

Sample	Tensile Strength (N/mm^2^)	% of EAB
Cp	0.96 ± 0.02	4.63 ± 0.64
Cp1	0.89 ± 0.04	17.94 ± 0.68
Cp2	1.22 ± 0.04	6.39 ± 0.53
Cp6	0.63 ± 0.05	10.51 ± 0.24

## Data Availability

The data presented in this study are available on request from the corresponding author.

## Supplementary Materials

The following are available online at https://www.mdpi.com/article/10.3390/polym14030463/s1, Figure S1: Green synthesized nanoparticles colour change observed from pale yellow to dark yellow then finally to reddish brown, Table S1: Swelling degree of films, Table S2: WVTR of films, Table S3: MRC of Scaffolds, Table S4: Degradation in Hank’s solution, Table S5: % of EAB of scaffolds, Table S6: Tensile strength of scaffolds.
