# Supplementary material for: Synthesis, Characterization and Physicochemical Properties of Biogenic Silver Nanoparticle-Encapsulated Chitosan Bionanocomposites
Source: Polymers (Basel). 2022 Jan 24;14(3):463. doi: 10.3390/polym14030463 (PMC8840532; doi:10.3390/polym14030463)
Supplement: Supplementary file 1 [file polymers-14-00463-s001.zip › polymers-1526771-supplementary.pdf]

polymers

MDPI

Article

Synthesis, Characterization and Physicochemical Properties of Biogenic Silver Nanoparticle-Encapsulated Chitosan Bionanocomposites

Sreelekha Ediyilyam[1], Mahesh M. L[1], Bini George[1]*, Sarojini Sharath Shankar[2,3]*, StanisławWacławek[4], Miroslav Černík[4]* and Vinod VelloraThekkae Padil[4]*

[1] Department of Chemistry, School of Physical Sciences, Central University of Kerala, Kerala, Kasaragod, Pin-671316, India; sreelekha.e469@gmail.com(S.E.);maheshml127@gmail.com (M.M.L.)
[2] Department of Biochemistry and Molecular Biology, School of Biological Sciences, Central University of Kerala, Kerala, Kasaragod, Pin-671316, India
[3] Department of Medicine, Thomas Jefferson University, Jefferson Alumni Hall, 1020 Locust Street, Philadelphia, PA19107, USA
[4] Institute for Nanomaterials, Advanced Technologies and Innovation (CXI), Technical University of Liberec (TUL), Studentská 1402/2, 461 17 Liberec, Czech Republic 1 461 17 Liberec, Czech Republic; stanislaw.waclawek@tul.cz
* Correspondence: binigeorgek@cukerala.ac.in (B.G.); sharathshankar82@gmail.com (S.S.S);miroslav.cernik@tul.cz (M.Č.); vinod.padil@tul.cz (V.V.T.P.); Tel.: +420-723-372-911 (V.V.T.P.)

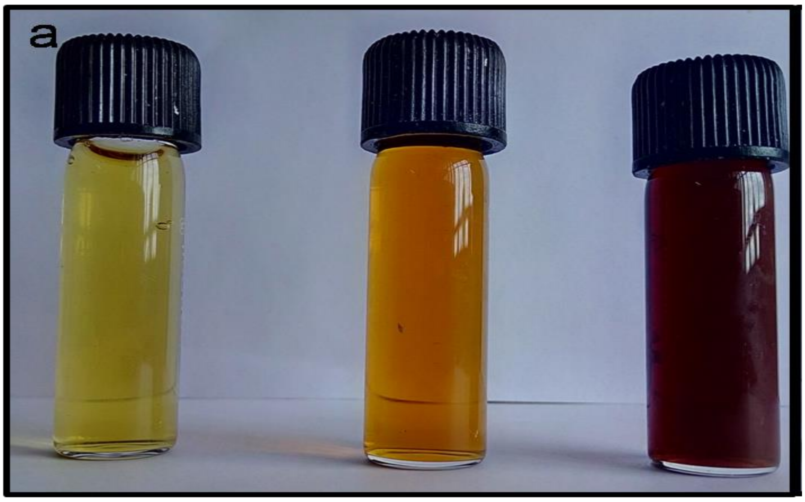

Figure S1. Green synthesized nanoparticles colour change observed from pale yellow to dark yellow then finally to reddish brown.

Table S1. Swelling degree of films.

Films	EDS of Scaffolds					
	x	y	z	Average	SD	SE
C	1160.7	1178	1170	1169.567	8.658137	1.698837
Cp	1327.65	1311	1326.9	1321.85	9.403856	1.770485
Cps	1228.23	1237.87	1224.87	1230.323	6.748076	1.499786
Cps1	1229.52	1245.45	1249.31	1241.427	10.49054	1.869986
Cps2	1250.05	1259.78	1251.31	1253.713	5.291525	1.328097
Cps3	1255.24	1263.98	1268.01	1262.41	6.528162	1.475145
Cps4	1281.85	1271	1293	1281.95	11.00034	1.914884

SD- standard deviation, SE- Standard error.

Table S2. WVTR of films.

Films	WVTR of Scaffolds					
	x	y	z	Average	SD	SE
C	60.51	61.02	60.76	60.76333	0.255016	0.291557
Cp	68.74	68.04	67.89	68.22333	0.453689	0.388882
Cps	67.65	67.51	68.09	67.75	0.302655	0.317624
Cps1	68.87	68.92	69.72	69.17	0.47697	0.398735
Cps2	69.76	69.43	69.01	69.4	0.375899	0.353977
Cps3	71.74	70.98	71.9	71.54	0.491528	0.404775
Cps4	75.68	75.88	76.21	75.92333	0.267644	0.298688

Table S3. MRC of Scaffolds.

Films	MRC of Scaffolds					
	x	y	z	Average	SD	SE
C	95.27	95.44	95.69	95.46667	0.211266	0.265371
Cp	97.3	97.39	97.64	97.44333	0.13167	0.2095
Cps	96.92	96.9	97.46	97.09333	0.284436	0.307915
Cps1	97.76	97.38	97.45	97.53	0.075056	0.158172
Cps2	97.81	97.99	98.38	98.06	0.207926	0.263266
Cps3	98.89	99.12	98.11	98.70667	0.507766	0.411406
Cps4	99.21	99.84	95.035	98.02833	2.426596	0.899369

Table S4. Degradation in Hank's solution.

Films	Hank's Degradation of Scaffolds					
	x	y	z	Average	SD	SE
C	926.12	910.87	937.25	924.7467	13.24351	2.101072
Cp	1698.2	1688.42	1677.5	1688.04	10.35523	1.857887
Cps	1542.69	1563.01	1552.75	1552.817	10.16016	1.840305
Cps1	1564.78	1574.64	1563.21	1567.543	6.195824	1.437106
Cps2	1568.56	1579.41	1571.37	1573.113	5.631166	1.370057
Cps3	1579.97	1589	1594.97	1587.98	7.551841	1.586594
Cps4	1598.2	1623.3	1634.9	1618.8	18.75926	2.500618

Table S5. % of EAB of scaffolds.

Films	% of EAB of Scaffolds					
	x	y	z	Average	SD	SE
C	4.6272	4.987	5.879	5.1644	0.64448	0.463494
Cp	17.9467	18.967	17.678	18.19723	0.680041	0.47611
Cps	6.3994	6.998	7.456	6.951133	0.529857	0.420261
Cps4	10.5161	10.789	10.999	10.76803	0.242132	0.284096

Table S6. Tensile strength (TS) of scaffolds

**Films**	**Tensile Strength of Scaffolds**					
	**x**	**y**	**z**	**Average**	**SD**	**SE**
C	0.9586	0.9284	0.9184	0.935133	0.020929	0.083524
Cp	0.8981	0.8145	0.8345	0.849033	0.043654	0.120629
Cps	1.2218	1.2645	1.2953	1.260533	0.03691	0.110921
Cps4	0.63295	0.7435	0.6543	0.676917	0.058643	0.139813
